# On-Clamp Versus Off-Clamp Robot-Assisted Partial Nephrectomy for Localized Renal Tumors: A Retrospective Single-Center Cohort Study

**DOI:** 10.3390/diagnostics16101543

**Published:** 2026-05-19

**Authors:** Stanila Stoeva-Grigorova, Simeon Marinov, Pavel Abushev, Plamen Kirilov, Doychin Nikolov, Turgay Kalinov, Nikola Kolev, Aleksandar Zlatarov, Lyuben Stoev, Deyan Dzhenkov

**Affiliations:** 1 Department of Pharmacology, Toxicology and Pharmacotherapy, Faculty of Pharmacy, Medical University of Varna, 9000 Varna, Bulgaria; 2 Clinic of Urology, University Hospital “St. Marina”, 9010 Varna, Bulgaria; dr.marinov.simeon95@gmail.com (S.M.); plamen.kirilov@mu-varna.bg (P.K.); 3 Department of Urology, Faculty of Medicine, Medical University of Varna, 9000 Varna, Bulgaria; pavel.abushev@mu-varna.bg (P.A.); dr.doychin.nikolov@gmail.com (D.N.); 4 Centre of Competence in Personalized Medicine, 3D and Telemedicine, Robotic-Assisted and Minimally Invasive Surgery “Leonardo da Vinci”, Medical University of Varna, 9000 Varna, Bulgaria; turgaykalinov92@gmail.com (T.K.); kolevsurgery@yahoo.com (N.K.); alekszlatarov@gmail.com (A.Z.); 5 Department of General and Operative Surgery, Faculty of Medicine, Medical University of Varna, 9000 Varna, Bulgaria; 6 First Clinic of Surgery, University Hospital “St. Marina”, 9010 Varna, Bulgaria; 7 Department of General and Clinical Pathology, Forensic Medicine and Deontology, Faculty of Medicine, Medical University of Varna, 9000 Varna, Bulgaria; lyubenstoev911@gmail.com (L.S.); ddzhenkov@gmail.com (D.D.); 8 Clinic of General and Clinical Pathology, University Hospital “St. Marina”, 9010 Varna, Bulgaria

**Keywords:** robot-assisted partial nephrectomy, on-clamp, off-clamp, renal cell carcinoma, nephron-sparing surgery, Trifecta, perioperative outcomes, renal function, RENAL score, complications

## Abstract

**Background:** Robot-assisted partial nephrectomy (RAPN) is an established nephron-sparing technique for localized renal tumors. It is performed using on-clamp (temporary renal artery clamping) or off-clamp (without hilar clamping) strategies. Comparative real-world evidence remains limited and is often confounded by non-randomized treatment allocation. **Methods:** This retrospective single-center study included 146 consecutive patients undergoing RAPN between 2020 and 2025. Patients were allocated to on-clamp (*n* = 108) or off-clamp (*n* = 38) groups based on tumor characteristics and intraoperative surgeon judgment. Perioperative, functional, and early oncological outcomes were analyzed. Tumor complexity was assessed using the RENAL nephrometry score. Surgical quality was evaluated using the Trifecta outcome (negative margins, warm ischemia time ≤25 min, and absence of Clavien–Dindo ≥III complications). **Results:** Off-clamp RAPN was more frequently applied in smaller tumors (*p* = 0.008), while RENAL scores were comparable between groups. Estimated blood loss was higher in the off-clamp group (260 ± 62 vs. 110 ± 35 mL; *p* < 0.0001), whereas transfusion rates and overall complication rates did not differ significantly. Trifecta achievement was similar between on-clamp and off-clamp RAPN (91.0% vs. 96.8%; *p* = 0.45). No significant differences were observed in early postoperative renal function (creatinine, hemoglobin, eGFR) or positive surgical margin rates. **Conclusions:** In this retrospective cohort, both on-clamp and off-clamp RAPN demonstrated comparable perioperative safety, functional outcomes, and early oncological efficacy. Differences in baseline tumor characteristics reflect selection bias rather than treatment effect. These findings support the feasibility of both techniques in appropriately selected patients, while highlighting the need for prospective comparative studies with adjustment for confounding factors.

## 1. Introduction

Robot-assisted partial nephrectomy (RAPN) was introduced into clinical practice in the early twenty-first century and has since become widely adopted as a nephron-sparing surgical approach for patients with clinically localized renal tumors [1,2,3,4]. Surgical intervention remains the cornerstone of curative treatment for localized renal cell carcinoma (RCC), and partial nephrectomy (PN) is recommended over radical nephrectomy (RN) in patients with clinical stage cT1 disease, based on evidence supporting preservation of renal function with comparable early oncological parameters [5,6]. Accumulating evidence suggests that RAPN provides favorable perioperative and functional outcomes, with oncological results comparable to those achieved with open and laparoscopic approaches [7,8,9,10]. In recent years, the indications for RAPN have expanded to include larger (cT2), anatomically complex, and bilateral renal tumors, as well as selected cases of local recurrence following prior nephron-sparing surgery [10,11]. These developments reflect increasing surgical experience and advances in robotic technology; however, outcomes may vary depending on tumor characteristics and patient selection.

Two principal technical strategies are currently distinguished in RAPN: the on-clamp and off-clamp approaches. In the on-clamp technique, temporary clamping of the renal artery is performed prior to tumor excision, facilitating hemostasis and providing stable operative conditions. However, this approach involves a period of warm ischemia, which may adversely affect postoperative renal function when prolonged [12,13,14,15]. Conversely, off-clamp RAPN is performed without temporary hilar clamping, with the aim of reducing ischemia-related renal injury [16,17,18]. This technique is often applied in selected patients with small renal masses (T1a, ≤4 cm) and in selected cases based on surgeon judgment and intraoperative assessment of tumor characteristics [10,13,15,17,19,20,21,22]. Previous studies comparing on-clamp and off-clamp RAPN have reported heterogeneous findings. While off-clamp approaches may reduce ischemia exposure, they have also been associated with increased intraoperative blood loss and greater technical complexity in some reports [19]. In addition, many studies include selected patient populations, and differences in tumor size and anatomical complexity between groups may influence outcomes.

In clinical practice, the choice of surgical strategy is not randomized and is typically guided by tumor characteristics, anatomical considerations, and surgeon experience [8]. In our tertiary referral center, off-clamp RAPN has been applied in selected patients, including some with moderately complex tumors; however, such observations should be interpreted cautiously due to the non-randomized nature of patient selection. Several randomized and retrospective studies have also questioned the clinical relevance of the presumed functional advantage of off-clamp RAPN when warm ischemia time is limited in optimized on-clamp procedures [15,20,23,24,25]. In this context, the comparative interpretation of outcomes between these techniques remains limited, particularly under routine clinical conditions. The present study aims to describe perioperative, functional, and early pathological parameters of on-clamp and off-clamp RAPN in a retrospective single-center cohort during the period 2020–2025. The study is exploratory and observational in nature and is not designed to establish superiority or equivalence between the two approaches, but rather to provide additional clinical data reflecting current surgical practice.

## 2. Materials and Methods

### 2.1. Study Design

This was a retrospective, non-randomized single-center cohort study conducted at the University Hospital “St. Marina”, Varna, Bulgaria. The study included patients who underwent RAPN between January 2020 and December 2025.

The choice between on-clamp and off-clamp RAPN was not randomized and was determined intraoperatively based on tumor anatomical characteristics, surgical judgment, and patient-specific clinical factors. Accordingly, confounding by indication cannot be excluded.

All patients were included consecutively during the study period to minimize selection bias. Procedures were performed within a single tertiary referral center by a limited number of experienced surgeons using a standardized surgical technique. Minor inter-surgeon variability in intraoperative decision-making, particularly regarding the use of renal hilar clamping, may have influenced treatment allocation.

Although the surgical protocol remained stable throughout the study period, a potential learning curve and temporal effect cannot be entirely excluded.

A total of 146 consecutive patients were included and allocated into two cohorts: on-clamp RAPN (*n* = 108) and off-clamp RAPN (*n* = 38). Importantly, all patients were initially considered part of a single operative cohort, and subgroup allocation reflects intraoperative surgical strategy rather than preoperative exclusion or selection. Of the 146 patients included in the final cohort, 132 had malignant renal tumors and 14 had benign histology. These 14 benign cases were not included in endpoint-specific analyses, including Trifecta assessment, and were not part of the evaluation of oncological outcomes due to the absence of applicable malignancy-specific endpoints.

For analytical purposes, additional outcome-specific subcohorts were defined. Histopathological analyses, including oncological margin status, were restricted to patients with malignant tumors. Similarly, Trifecta assessment was performed only in patients with malignant pathology and complete perioperative datasets. Positive surgical margins were analyzed exclusively in patients with malignant renal tumors (*n* = 132) to ensure consistency of oncological endpoint definition. All 146 patients were included in the overall cohort. However, for oncological endpoint analyses (Trifecta and margin status), only patients with histologically confirmed malignant tumors were considered. Patients with benign pathology were excluded from oncological endpoint analyses only after final histopathological classification.

ο Inclusion criteria:
  ‑ Age ≥18 years;
  ‑ Radiologically confirmed renal tumor deemed suitable for PN;
  ‑ Complete preoperative laboratory and imaging data available;
  ‑ Documented RENAL nephrometry score.
ο Exclusion criteria:
  ‑ Incomplete clinical or laboratory data;
  ‑ Missing RENAL score documentation;
  ‑ Non-PN procedures (converted to RN).

Benign and non-RCC lesions excluded from oncological endpoint analyses due to lack of applicability of Trifecta criteria.

**Figure 1:**
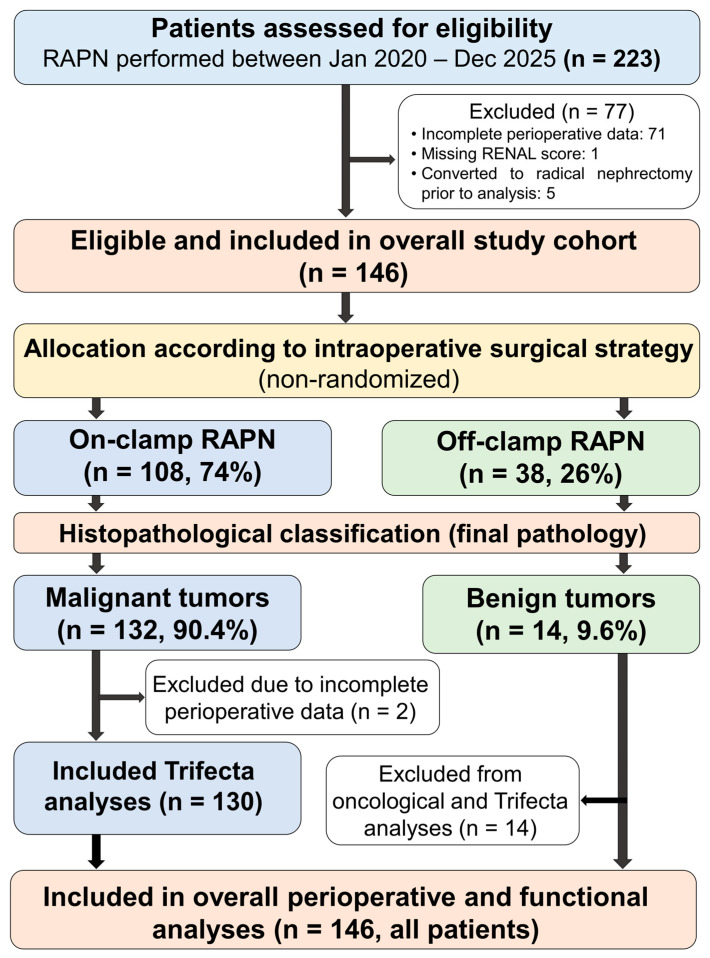
Flow diagram of patient selection.

The study is reported in accordance with the STROBE guidelines, and all limitations related to confounding by indication and non-randomized design are explicitly acknowledged (Supplementary Table S1) [26]. The flow of patient selection and reasons for exclusion are presented in Figure 1.

#### 2.1.1. Study Size

No formal sample size calculation was performed due to the retrospective and exploratory nature of the study. The study cohort represents all consecutive patients undergoing RAPN at our institution during the predefined study period (2020–2025), reflecting real-world clinical practice.

#### 2.1.2. Bias

Several sources of bias are inherent to the study design. Most importantly, confounding by indication is present, as surgical strategy (on-clamp vs. off-clamp) was determined by tumor complexity and intraoperative judgment rather than randomization. Selection bias was minimized by consecutive patient inclusion; however, baseline imbalance in tumor characteristics between groups remains. Information bias is limited due to the use of standardized institutional records and predefined outcome definitions.

### 2.2. Demographic and Clinical Variables

Data were collected on age, sex, surgical approach (on-clamp vs. off-clamp), and warm ischemia time (for the on-clamp cohort). Patients were stratified into three age categories: <45 years, 45–60 years, and >60 years. However, detailed structured comorbidity indices (e.g., Charlson Comorbidity Index) were not systematically available in the dataset.

Surgical quality was evaluated using the Trifecta criterion, defined as a composite surgical quality endpoint consisting of negative surgical margins, warm ischemia time ≤ 25 min, and absence of Clavien–Dindo grade ≥ III complications within 3 months postoperatively [27,28].

### 2.3. Surgical Technique

All procedures were performed using the da Vinci Xi robotic surgical system via a standardized transperitoneal approach, with patients positioned in a lateral decubitus position with table flexion. Following induction of general anesthesia and sterile preparation of the operative field, pneumoperitoneum was established, and four robotic trocars along with one assistant port were placed under direct visualization. The robotic system was subsequently docked.

Colonic mobilization was performed along the line of Toldt to achieve medial reflection and expose the retroperitoneal space. Gerota’s fascia was incised, and the kidney was mobilized to allow adequate exposure of the tumor and surrounding parenchyma. Tumor localization was based on preoperative cross-sectional imaging; intraoperative ultrasound was not routinely employed.

In the on-clamp RAPN group, meticulous hilar dissection was performed to identify and isolate the renal artery. Temporary arterial clamping was achieved using a robotic vascular clamp prior to tumor excision, with warm ischemia time actively minimized and typically maintained within clinically accepted limits. Venous clamping was not routinely performed. In the off-clamp RAPN group, tumor resection was carried out without hilar clamping, at the discretion of the operating surgeon. This approach was more frequently applied in patients with smaller tumors and predominantly exophytic lesions, based on intraoperative assessment and surgeon preference. Hilar dissection in these cases was either omitted or limited to anatomical identification without vascular control.

Tumor excision in both groups was performed using a sharp, margin-oriented resection technique, aiming to achieve negative surgical margins while preserving maximal functional renal parenchyma. The resection strategy corresponded predominantly to an enucleoresection approach. In selected cases, intraoperative frozen section analysis was used to confirm margin status.

Hemostasis was achieved using a combination of bipolar coagulation and application of oxidized regenerated cellulose (Surgicel^®^). Renal reconstruction (renorrhaphy) was performed in a standardized two-layer fashion, consisting of an inner layer for closure of the collecting system and deep parenchymal vessels when required, followed by an outer cortical layer using a sliding-clip technique to ensure adequate compression and hemostasis. No significant variations in reconstruction technique were observed between the two groups.

Intraoperative parameters included estimated blood loss (EBL, mL), warm ischemia time (minutes), and intraoperative complications.

All procedures were performed by experienced robotic surgeons in a high-volume tertiary referral center using a standardized operative workflow, although minor variability in intraoperative decision-making—particularly regarding vascular control—was permitted based on individual tumor characteristics.

### 2.4. Perioperative Outcomes and Complications

Perioperative complications were recorded and classified according to the Clavien–Dindo grading system (grades 0–V), an internationally accepted standard for grading surgical complications based on the level of therapeutic intervention required [29,30]. Perioperative complications were recorded within 30 days after surgery, whereas the Clavien–Dindo grade ≥ III component of the Trifecta outcome was additionally assessed within 3 months postoperatively in accordance with published Trifecta definitions in partial nephrectomy literature. This extended follow-up interval was used specifically to capture clinically relevant delayed major complications potentially occurring beyond the standard 30-day perioperative period. Data collection included all postoperative events, including minor and major complications, readmissions, reinterventions, urinary leakage, postoperative bleeding (including pseudoaneurysm), and infectious complications. Conversion events (conversion to open surgery, RN, or intraoperative switch of clamping strategy) were also documented when applicable. All data were obtained from prospective chart review and institutional medical records.

Renal function was assessed using serum creatinine (Cr, μmol/L), hemoglobin (Hb, g/L), and estimated glomerular filtration rate (eGFR, mL/min/1.73 m^2^), calculated using the CKD-EPI equation. Cr and Hb were assessed at two predefined time points: preoperatively (within 24 h before surgery) and early postoperatively (48–72 h after surgery, before discharge). Because preoperative eGFR values were not systematically available for the entire cohort, ΔeGFR was not used as a primary comparative endpoint. Instead, renal functional change was primarily assessed using paired change in serum Cr (ΔCr), which was consistently available for comparative analysis between surgical groups. Renal functional outcomes were analyzed both as absolute postoperative values and as changes from baseline for serum Cr measurements.

### 2.5. Tumor Anatomical Assessment

Tumor anatomical complexity was quantified using the RENAL nephrometry score, which includes:

ο R—Radius (tumor size);
ο E—Exophytic/endophytic properties;
ο N—Nearness to the collecting system or sinus;
ο A—Anterior/posterior location;
ο L—Location relative to the polar lines.

This scoring system enables standardized assessment of surgical complexity and is widely employed in the planning of nephron-sparing procedures [31,32].

### 2.6. Histopathology and Staging

Histological subtype was determined for all resected tumors. Pathological staging was performed according to the TNM classification of the American Joint Committee on Cancer/Union for International Cancer Control [33,34,35]:

ο T1a: tumors ≤ 4 cm;
ο T1b: tumors > 4–7 cm;
ο T2a: tumors > 7–10 cm;
ο T2b: tumors > 10 cm;
ο T3a: tumor extension into perirenal fat or renal vein without invasion beyond Gerota’s fascia.

Tumor anatomical complexity and oncological stage represent distinct and non-comparable clinical dimensions. The RENAL nephrometry score was used exclusively as a preoperative anatomical complexity metric, whereas TNM staging was determined postoperatively based on histopathological examination. For analytical purposes, benign tumors and non-RCC malignancies were grouped as non-RCC lesions. These cases were excluded from Trifecta and oncological endpoint analyses due to the absence of applicable malignancy-specific endpoints.

### 2.7. Statistical Analysis

Statistical analyses were performed using GraphPad Prism version 9.0. Continuous variables were assessed for normality using the Shapiro–Wilk test. Normally distributed data are presented as mean ± standard deviation and were compared using unpaired Student’s *t*-test (with Welch’s correction when appropriate). Non-normally distributed variables were analyzed using the Mann–Whitney U test. Categorical variables were compared using χ^2^ or Fisher’s exact test. Fisher’s exact test was applied when expected cell counts were <5, whereas χ^2^ test was used for larger samples. For all endpoint-specific analyses, the denominator was defined according to data availability for each outcome, resulting in minor variations in sample size across analyses. Operative time was analyzed in a complete-case subset due to missing data and was reported descriptively without performing between-group statistical comparisons.

Given the observational and non-randomized nature of the study, no formal propensity score matching or multivariable regression models were applied. Instead, subgroup analyses and baseline comparisons were used to assess potential confounding between surgical groups. No multivariable regression or propensity score methods were applied due to sample size limitations and the exploratory nature of the study. Therefore, the results should be interpreted as descriptive rather than causal.

Renal functional outcomes were additionally analyzed using paired change-from-baseline serum creatinine values (ΔCr) to partially account for baseline differences between groups. Because preoperative eGFR values were not systematically available for the entire cohort, ΔeGFR analysis was not performed.

A two-sided *p*-value < 0.05 was considered statistically significant. Adjustment for multiple comparisons was not applied due to the exploratory nature of the study.

Missing data were handled by complete-case analysis. Only patients with complete datasets for the respective variables were included in each analysis.

## 3. Results

### 3.1. Baseline and Demographic Characteristics

During the period 2020–2025, a total of 146 patients underwent RAPN, aged between 37 and 90 years. Although the overall mean age was 60.67 ± 12.71 years, patients older than 60 years represented the largest categorical age subgroup—77 cases (52.7%), followed by those aged 45–60 years—55 patients (37.7%), and patients younger than 45 years—14 cases (9.6%).

Regarding surgical strategy, on-clamp RAPN was performed in 108 patients (74%), with a mean clamping time of 15.39 ± 4.08 min, whereas off-clamp RAPN was applied in 38 patients (26%). Operative time data were available only in a subset of patients and are therefore reported descriptively without formal statistical comparison between groups. Patients older than 60 years constituted the largest subgroup in both cohorts, with 51/108 (47.2%) in the on-clamp group and 26/38 (68.4%) in the off-clamp group. Patients aged 45–60 years accounted for 41.7% and 26.3%, respectively, while those under 45 years comprised 11.1% and 5.3%. Percentages were calculated relative to the number of patients within each surgical group. The distribution of patients by age categories (<45, 45–60, and >60 years) did not differ statistically between on-clamp and off-clamp RAPN (χ^2^ = 5.16, df = 2, *p* = 0.076).

**Figure 2:**
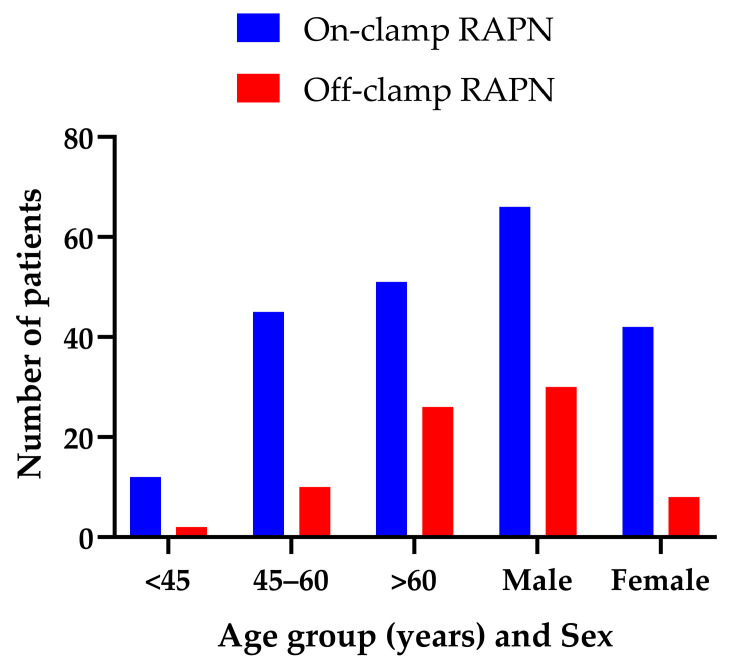
Distribution of Patients Undergoing On-clamp or Off-clamp RAPN by Age and Sex.

With respect to sex distribution, males predominated—96 patients (65.8%) versus 50 females (34.2%). In the on-clamp group, 66 patients (61.1%) were male, whereas in the off-clamp group, 30 patients (78.9%) were male. The difference between groups was statistically significant (χ^2^ = 3.97; df = 1; *p* = 0.046; Fisher’s exact *p* = 0.050), with a higher proportion of males in the off-clamp cohort (Figure 2).

Baseline demographic, tumor, and perioperative characteristics of patients stratified by surgical approach are summarized in Table 1.

These baseline differences in tumor characteristics between surgical groups should be interpreted as evidence of non-random treatment allocation rather than true clinical equivalence at baseline.

### 3.2. Tumor Characteristics

The mean tumor size was significantly higher in the on-clamp cohort compared to the off-clamp cohort (4.20 ± 1.01 cm vs. 3.7 ± 1.05 cm, respectively; median 4.0 cm in both groups). The Mann–Whitney U test demonstrated a statistically significant difference between groups (U = 1486, *p* = 0.008), indicating that off-clamp RAPN was more frequently applied in patients with smaller renal masses. Tumor distribution analysis further confirmed that smaller tumors were more often managed using the off-clamp approach, whereas larger lesions were predominantly treated with the on-clamp technique (Table 2).

With respect to morphological characteristics, exophytic growth was observed in slightly more than half of the patients (56.2%). The remaining tumors were predominantly endophytic. Off-clamp RAPN was more frequently employed in tumors with smaller size and exophytic characteristics, while overall RENAL score did not differ between groups.

The anatomical complexity of renal tumors, assessed using the RENAL nephrometry score, demonstrated a predominance of lesions with moderate complexity. TNM stage was analyzed postoperatively. In the on-clamp RAPN group, tumors with RENAL score 6 (44 patients, 40.7%) and 7 (24 patients, 22.2%) were most common. Similarly, in the off-clamp RAPN group, tumors with comparable anatomical complexity predominated, with RENAL score 6 identified in 12 patients (31.6%) and score 7 in 10 patients (26.3%). Tumors with higher RENAL scores (8–9) were relatively rare and occurred mainly in the on-clamp group. No statistically significant difference in tumor complexity between the two groups was observed. The median RENAL score was 6.0 in both on-clamp and off-clamp RAPN. Mann–Whitney U test (two-tailed) showed no statistically significant difference (U = 1831; exact *p* = 0.304).

Regarding TNM stage, tumors were most frequently diagnosed at early stages T1a (68 patients, 46.6%) and T1b (53 patients, 36.3%). In the on-clamp RAPN group, T1a tumors were observed in 45 patients (41.7%), and T1b in 44 patients (40.7%). In the off-clamp RAPN group, T1a tumors were identified in 23 patients (60.5%), and T1b in 9 patients (23.7%). More advanced stages (T2a, T2b, and T3a) were rare in both groups. Due to the limited number of patients with advanced disease, TNM stage was analyzed after grouping into T1 and ≥T2 categories. In both surgical groups, T1 tumors predominated—89 patients (90.8%) in the on-clamp and 32 patients (94.1%) in the off-clamp group. Stages ≥T2 were observed in 9 (9.2%) and 2 (5.9%) patients, respectively. Comparison using two-tailed Fisher’s exact test revealed no statistically significant difference (*p* = 0.728).

Histological analysis demonstrated a predominance of clear cell RCC (93 patients, 63.7%). It predominated in the on-clamp group (75 patients, 69.4%), whereas in the off-clamp group it accounted for 18 patients (47.4%). Papillary carcinoma was observed in 17 patients (11.6%)—7 patients (6.5%) in the on-clamp and 10 (26.3%) in the off-clamp group. Chromophobe carcinoma was identified in 11 patients (7.5%), with 9 (8.3%) in the on-clamp and 2 (5.3%) in the off-clamp cohort. Other rare and benign histological variants (cystic nephroma, angiomyolipoma, oncocytoma, Ewing sarcoma, etc.) were observed in a limited number of cases. Detailed anatomical, staging, and histological tumor characteristics according to surgical approach are presented in Table 3. Histological subtype and TNM stage represent postoperative pathological outcomes.

Overall, these findings demonstrate a clear pattern of treatment allocation, with off-clamp RAPN being preferentially applied in tumors with lower anatomical complexity and smaller size, consistent with confounding by indication in this retrospective cohort.

### 3.3. Perioperative Outcomes

The EBL was significantly higher in the off-clamp group (260.0 ± 62.0 mL) compared to the on-clamp group (110.0 ± 35.0 mL). The mean difference between groups was 150.0 ± 10.61 mL (95% CI: 128.7–171.3 mL; t = 14.14; df = 45.6; *p* < 0.0001). The F-test indicated unequal variances between groups (F = 3.14). Despite higher intraoperative blood loss in the off-clamp cohort, transfusion rates were low and comparable between groups (5/108 vs. 1/38; Fisher’s exact test, *p* > 0.05). Specifically, 5 patients (4.6%) in the on-clamp cohort and 1 patient (2.6%) in the off-clamp cohort required blood transfusions.

Perioperative complications were classified according to the Clavien–Dindo system. Table 4 presents the full distribution of complications (grades 0–V), whereas clinically significant complications (≥Grade II) are analyzed separately in Table 5. Grade II complications occurred in 13 patients (8.9%), while grade IIIa and IIIb complications were rare, occurring in 2 (1.4%) and 1 (0.7%) patients, respectively. When stratified by surgical approach, ≥Grade II complications were observed in 15/108 patients (13.9%) in the on-clamp group and in 1/38 patients (2.6%) in the off-clamp group. This difference did not reach statistical significance (Fisher’s exact test, *p* = 0.0705). The corresponding odds ratio was 5.97 (95% CI: 0.91–64.67, Baptista–Pike method). For higher-grade events, ≥Grade III complications occurred in 3/108 patients (2.8%) in the on-clamp group and in 0/38 patients (0%) in the off-clamp group, without statistical significance (Fisher’s exact test, *p* = 0.5679).

Within the study cohort, postoperative bleeding occurred in two patients, while infectious complications were observed in five cases. No instances of urinary leakage or renal pseudoaneurysm were identified. No intraoperative conversions (to open surgery or RN), readmissions, or unplanned reinterventions were recorded within the 30-day postoperative period. These findings complement the Clavien–Dindo classification and provide a more granular characterization of perioperative complications following RAPN.

To enhance methodological transparency, each component of the Trifecta endpoint was additionally analyzed separately. Negative surgical margins were achieved in 128 of 130 evaluable patients (98.5%), while absence of Clavien–Dindo grade ≥ III complications was observed in 126 of 130 patients (96.9%). Warm ischemia time analysis demonstrated a mean duration of 15.3 min in the on-clamp group, remaining well below the predefined threshold of 25 min, whereas off-clamp procedures inherently demonstrated zero ischemia time due to the absence of hilar clamping.

Trifecta analysis was performed in 130 evaluable patients with malignant renal tumors (Table 6), following exclusion of patients with benign histology and two cases with incomplete perioperative data. Trifecta was achieved in 90 of 99 patients (91.0%) in the on-clamp group and in 30 of 31 patients (96.8%) in the off-clamp group. No statistically significant difference was observed between groups (Fisher’s exact test, *p* = 0.4498). The odds ratio for Trifecta achievement (on-clamp vs. off-clamp) was 0.326 (95% CI 0.029–2.195, Baptista–Pike method). The wide confidence interval reflects substantial statistical imprecision and should therefore be interpreted cautiously.

### 3.4. Laboratory and Functional Parameters

Positive surgical margins were observed in 2 patients in the on-clamp group and in 0 patients in the off-clamp group. For consistency of oncological endpoint analysis, margins were evaluated within the malignant cohort. Accordingly, these events correspond to 2/100 malignant on-clamp cases (2.0%) and 0/32 malignant off-clamp cases (0.0%), calculated within the malignant cohort (*n* = 132). The difference between groups was not statistically significant. All cases with positive margins were confirmed on final histopathological examination. No cases of local tumor recurrence were observed during the early follow-up period in either surgical group.

Preoperative serum Cr was higher in the off-clamp group (90.8 ± 21.4 μmol/L) compared to on-clamp (77.4 ± 18.6 μmol/L). The difference was statistically significant (13.4 ± 3.91 μmol/L; 95% CI: 5.58–21.22 μmol/L; *p* = 0.0011). Variance comparison was not significant (F = 1.324; *p* = 0.270). Postoperatively, Cr increased to 85.9 ± 20.2 μmol/L in on-clamp and 91.2 ± 22.1 μmol/L in off-clamp patients. The intergroup difference was not statistically significant (5.3 ± 4.08 μmol/L; 95% CI: −2.86 to 13.46 μmol/L; *p* = 0.199; F = 1.197; *p* = 0.473). Due to non-normal distribution of paired differences, within-group analyses were performed using the Wilcoxon matched-pairs signed-rank test, whereas intergroup comparison of ΔCr values was performed using the Mann–Whitney U test. The on-clamp cohort demonstrated a significant postoperative increase in serum creatinine, with a median ΔCr of 6.0 μmol/L (95% CI: 3.0–8.0 μmol/L; *p* < 0.0001). In contrast, the off-clamp group showed no statistically significant postoperative deterioration in renal function, with a median ΔCr of 1.0 μmol/L (95% CI: 0.2–2.0 μmol/L; *p* = 0.318). Direct comparison of change-from-baseline values demonstrated significantly greater postoperative creatinine elevation in the on-clamp cohort compared with off-clamp RAPN (median difference: −5.0 μmol/L; Hodges–Lehmann estimate: −6.0 μmol/L; 95% CI: −9.0 to −2.0 μmol/L; Mann–Whitney U test, *p* = 0.0009).

Functional analysis of renal outcomes demonstrated comparable early postoperative eGFR values: 86.5 ± 17.8 mL/min/1.73 m^2^ in on-clamp and 80.8 ± 19.6 mL/min/1.73 m^2^ in off-clamp patients. The difference (B—A) was −5.7 ± 3.61 mL/min/1.73 m^2^ (95% CI: −12.92 to 1.53), without statistical significance (*p* = 0.120). Variance comparison was likewise non-significant (F = 1.212; *p* = 0.444). Because preoperative eGFR values were incompletely available, postoperative eGFR findings should be interpreted as descriptive early functional measurements rather than true change-from-baseline functional outcomes.

Preoperative Hb levels were similar between groups: 140.6 ± 12.4 g/L in on-clamp and 137.0 ± 13.1 g/L in off-clamp RAPN. The difference between groups (B—A) was −3.60 ± 2.44 g/L (95% CI: −8.47 to 1.27 g/L; *p* = 0.145). Variance comparison was not significant (F = 1.116, *p* = 0.651). Postoperatively, Hb decreased to 126.6 ± 13.8 g/L in on-clamp and 125.9 ± 12.9 g/L in off-clamp patients. The difference remained non-significant (−0.70 ± 2.48 g/L; 95% CI: −5.64 to 4.24 g/L; *p* = 0.779; F = 1.144; *p* = 0.654).

**Figure 3:**
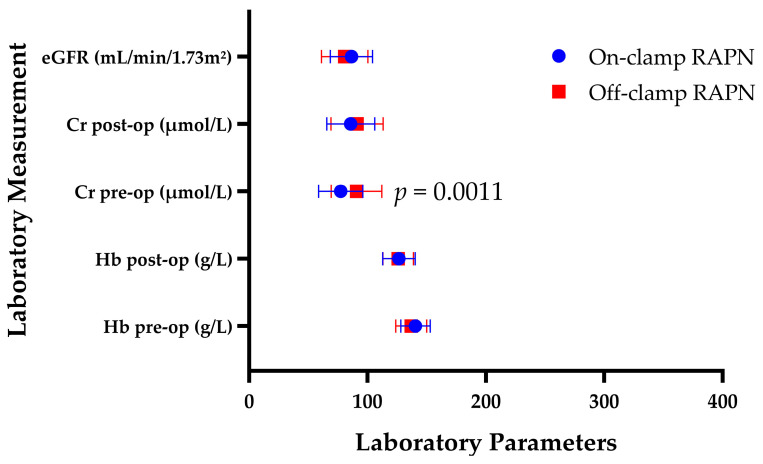
Laboratory outcomes in on-clamp versus off-clamp RAPN, including serum Cr (μmol/L), Hb (g/L), and early postoperative eGFR (mL/min/1.73 m^2^). Serum Cr and Hb were assessed both preoperatively and at the early postoperative timepoint (48–72 h after surgery), whereas eGFR analysis reflects postoperative measurements only due to incomplete availability of paired preoperative eGFR data. Data are presented as mean ± standard deviation (SD). Between-group comparisons were performed using unpaired *t*-tests with Welch’s correction. *p* < 0.05 indicates statistical significance.

Figure 3 illustrates the distribution of preoperative and early postoperative laboratory parameters in patients undergoing on-clamp and off-clamp RAPN.

## 4. Discussion

This retrospective study provides real-world data on the safety and efficacy of on-clamp and off-clamp RAPN in patients with localized renal tumors. The demographic characteristics of the patients were generally similar between the groups. Although sex distribution differed slightly between the groups (*p* = 0.046), this imbalance did not translate into clinically meaningful differences in perioperative or functional outcomes. Importantly, the observed sex imbalance is most likely incidental and reflects the non-randomized treatment allocation process rather than a biologically or anatomically driven selection mechanism.

A statistically significant and clinically relevant baseline imbalance was observed in tumor characteristics between the two surgical groups. Off-clamp RAPN was more frequently applied to smaller tumors, while overall RENAL scores were comparable, indicating similar anatomical complexity. In addition, these tumors were more often exophytic and associated with lower surgical complexity. This pattern reflects confounding by indication, as treatment allocation was primarily driven by tumor anatomy and surgeon judgment rather than randomization [36]. Therefore, the two cohorts should not be considered fully baseline-comparable with respect to tumor complexity, and all intergroup comparisons should be interpreted as descriptive and hypothesis-generating. TNM staging, as a postoperative pathological parameter, cannot account for preoperative selection bias.

Despite the higher intraoperative blood loss observed in the off-clamp cohort, transfusion rates remained comparable, indicating that blood loss was clinically manageable with both surgical approaches. Overall, the perioperative complication profile was similar between groups, indicating a comparable safety profile, although interpretation is limited by baseline differences in tumor complexity. Oncological radicality, assessed by positive surgical margins, also appeared comparable between techniques, suggesting that both approaches can be applied safely in selected clinical settings. It should be emphasized that positive surgical margins were analyzed exclusively within the malignant cohort to ensure methodological consistency across oncological endpoints. Importantly, margin status was analyzed exclusively within the malignant cohort to ensure methodological consistency. These findings are in line with previous meta-analyses reporting similar transfusion and complication profiles despite differences in blood loss between techniques [22,23].

Severe complications were rare and were observed predominantly in the on-clamp group, consistent with previous studies that did not demonstrate statistically significant differences in Clavien–Dindo ≥ III complications between techniques, although some reports have described a numerical trend favoring the off-clamp approach [22,24]. Surgical site infections (SSIs) are a recognized postoperative complication following partial nephrectomy, including RAPN. Although overall incidence is low in minimally invasive approaches, factors such as prolonged operative time, intraoperative blood loss, comorbidities (e.g., diabetes), and tumor complexity may increase SSI risk. In our cohort, no severe SSIs were observed, consistent with previous reports indicating rates below 5% in robotic-assisted procedures. Preventive strategies include meticulous aseptic technique, judicious use of perioperative antibiotics, and careful wound management. Awareness and early recognition of SSIs are essential, as they may prolong hospitalization and affect patient recovery [37,38].

Trifecta achievement was consistently high in both groups (91.0% for on-clamp and 96.9% for off-clamp RAPN), with no statistically significant difference. These findings are in agreement with other retrospective studies, in which the choice of clamping strategy did not significantly influence Trifecta outcomes [39,40]. It should be acknowledged that Trifecta, although widely used as a composite indicator of surgical quality in partial nephrectomy, is not fully symmetric between on-clamp and off-clamp techniques. Specifically, the warm ischemia time component inherently favors off-clamp procedures, in which ischemia time is zero by definition. This structural characteristic may lead to a relative overestimation of Trifecta achievement in the off-clamp group when used as a comparative endpoint. Accordingly, interpretation of Trifecta in comparative analyses between clamping strategies should be performed with caution.

From a methodological perspective, Trifecta incorporates parameters that are directly influenced by the surgical technique; therefore, it does not represent a fully technique-independent endpoint. In this context, Trifecta achievement in the off-clamp cohort reflects a modified application of the original definition. To address this limitation, the present study additionally evaluated each component of the Trifecta separately, allowing a more granular interpretation of perioperative quality.

Laboratory and functional outcomes were comparable between groups. Although preoperative creatinine levels were higher in the off-clamp group, postoperative creatinine, hemoglobin, and eGFR values did not differ significantly. These findings suggest no early functional disadvantage associated with either surgical approach within the limitations of available renal function data.

These results are consistent with contemporary trends in nephron-sparing surgery, where early detection of small renal masses enables safe application of RAPN in most clinical settings [41]. More than 70% of renal cell carcinomas are diagnosed at early stages, and PN for tumors < 4 cm is associated with excellent 5-year cancer-specific survival. RCC remains the predominant malignant renal tumor (~90%), whereas other histological subtypes are relatively uncommon. Modern imaging modalities, including ultrasound, computed tomography, and magnetic resonance imaging, play a central role in early detection and surgical planning [42,43].

Several limitations should be acknowledged. The retrospective single-center design is associated with inherent selection bias, as treatment allocation was determined by tumor characteristics and intraoperative judgment, resulting in non-random group assignment. Baseline comparisons confirmed that off-clamp RAPN was more frequently applied in smaller and less complex tumors. No multivariable adjustment was performed, limiting the ability to fully account for baseline imbalances. The relatively small size of the off-clamp cohort and the limited number of events further reduce statistical power. Consequently, the confidence intervals for some comparative estimates, including Trifecta achievement, were wide and do not allow reliable inference regarding the magnitude or direction of potential differences between surgical techniques. Operative time data were incomplete and could not be analyzed. In addition, surgeon-dependent factors and potential learning curve effects cannot be excluded. Long-term functional and oncological outcomes were not systematically available; therefore, outcome assessment is limited to early perioperative and pathological parameters, including histological subtype and surgical margin status. The Trifecta endpoint, although widely used, is not fully technique-independent, as the absence of ischemia in off-clamp procedures inherently favors its achievement. For this reason, individual Trifecta components were also analyzed separately to allow a more detailed interpretation of perioperative outcomes. Finally, the use of ASA score provides only a general estimate of patient status, and exclusion of cases with incomplete data may have introduced additional bias.

## 5. Conclusions

This retrospective single-center cohort study compared on-clamp and off-clamp RAPN in patients with localized renal tumors under real-world, non-randomized conditions. The choice of surgical strategy was driven by tumor anatomy and surgeon judgment, resulting in a clear selection pattern, with off-clamp procedures more frequently applied in smaller and less complex tumors.

Within these inherent baseline differences, both techniques demonstrated acceptable short-term perioperative safety profiles and oncopathological outcomes within the limitations of this non-randomized cohort. A higher intraoperative blood loss was observed in the off-clamp group, while other perioperative parameters, complication rates, and composite quality endpoints showed no statistically significant differences.

However, due to the retrospective design, absence of randomization, and lack of adjusted analyses, the findings should be interpreted as associative rather than causal. Confounding by indication, imbalance in tumor complexity, and the limited size of the off-clamp cohort substantially restrict the ability to draw definitive comparative conclusions.

Overall, the study supports the feasibility of both approaches in routine clinical practice and highlights that off-clamp RAPN is preferentially used in anatomically less complex cases. Further prospective, ideally multicenter studies with standardized adjustment for baseline differences are required to more robustly define comparative effectiveness between the two techniques.

## Figures and Tables

**Table 1:** Baseline demographic, tumor, and perioperative characteristics.

Variable	On-Clamp RAPN (*n* = 108)	Off-Clamp RAPN (*n* = 38)	Overall (*n* = 146)	*p*-Value
Age (years), mean ± SD	59.02 ± 11.41	65.79 ± 11.65	60.67 ± 12.71	0.076
Sex (Male/Female)	66/42	30/8	96/50	0.046
ASA score (% within group)	2: 38 (35.2%) 3: 62 (57.4%) 4: 8 (7.4%)	2: 15 (39.5%) 3: 21 (55.3%) 4: 2 (5.2%)	2: 53 (36.3%) 3: 83 (56.8%) 4: 10 (6.9%)	—
Tumor size (cm), mean ± SD	4.20 ± 1.01	3.70 ± 1.05	—	—
RENAL score, median (IQR)	6 (5–7)	6 (5–7)	6 (5–7)	—
Laterality (L/R)	52/56	19/19	71/75	—
Endophytic tumors	46 (42.6%)	18 (47.4%)	64 (43.8%)	—
Operative time (min), median (IQR) ^†^	122 (90–150)	118 (85–140)	121 (88–148)	—
Clamping time (min ± SD)	15.39 ± 4.08	NA	NA	—
Hospital stay (days), median (IQR)	5 (4–7)	4 (3–6)	5 (4–7)	—
Positive surgical margin	2 (1.9%)	0 (0%)	2 (1.4%)	—

Variables are presented as mean ± SD or median (IQR) according to distribution; RENAL score is presented as a non-normally distributed variable; ^†^ Operative time was available in a subset of patients (on-clamp: *n* = 91; off-clamp: *n* = 36). Values are presented descriptively only, and no formal between-group statistical comparison was performed due to incomplete data availability.

**Table 2:** Distribution of Renal Tumors by Size and Type of RAPN (On-clamp vs. Off-clamp) in 146 Patients.

Tumor Size (cm)	Number of Patients (*n*)	% of Total	On-Clamp RAPN (*n*)	% of On-Clamp	Off-Clamp RAPN (*n*)	% of Off-Clamp
2	8	5.5	3	2.8	5	13.2
3	35	24.0	22	20.4	13	34.2
4	55	37.7	44	40.7	11	28.9
5	39	26.7	31	28.7	8	21.1
6	7	4.8	6	5.6	1	2.6
7	1	0.7	1	0.9	0	0
8	1	0.7	1	0.9	0	0
Total	146	100	108	100	38	100

**Table 3:** Tumor characteristics, pathological stage, and histological findings stratified by surgical approach (on-clamp vs. off-clamp RAPN) in 146 patients.

Variable	Category	Total (*n* = 146)	% of Total	On-Clamp RAPN (*n* = 108)	% of On-Clamp	Off-Clamp RAPN (*n* = 38)	% of Off-Clamp
RENAL score	4	12	8.2	6	5.6	6	15.8
	5	31	21.2	23	21.3	8	21.1
	6	56	38.4	44	40.7	12	31.6
	7	34	23.3	24	22.2	10	26.3
	8	12	8.2	11	10.2	1	2.6
	9	1	0.7	0	0	1	2.6
TNM stage *	T1a	68	46.6	45	41.7	23	60.5
	T1b	53	36.3	44	40.7	9	23.7
	T2a	3	2.1	2	1.9	1	2.6
	T2b	0	0	0	0	0	0
	T3a	8	5.5	7	6.5	1	2.6
Histology *	Clear cell RCC	93	63.7	75	69.4	18	47.4
	Papillary RCC	17	11.6	7	6.5	10	26.3
	Chromophobe RCC	11	7.5	9	8.3	2	5.3
	Cystic nephroma	9	6.2	6	5.6	3	7.9
	Angiomyolipoma	7	4.8	5	4.6	2	5.3
	Oncocytoma	4	2.7	3	2.8	1	2.6
	Ewing sarcoma	2	1.4	2	1.9	0	0
	Liposarcoma	1	0.7	1	0.9	0	0
	Leiomyoma	1	0.7	1	0.9	0	0
	Metastatic breast carcinoma	1	0.7	1	0.9	0	0

* Percentages in the “Total” column refer to the entire study population (*n* = 146), while percentages in the on-clamp and off-clamp columns refer to subgroup distributions. RENAL score represents a preoperative imaging-based assessment applied to all patients. TNM stage and histological subtype are reported only for malignant tumors and are therefore calculated using the malignant subset as denominator. Rare histological entities may appear proportionally inflated within subgroups due to low absolute numbers.

**Table 4:** Clavien–Dindo complications stratified by surgical approach.

Clavien–Dindo Grade	*n* (Total)	% Total Cohort	On-Clamp RAPN (*n* = 108)	% On-Clamp	Off-Clamp RAPN (*n* = 38)	% Off-Clamp
Grade 0 (No complication)	84	57.5	58	53.7	26	68.4
Grade I	46	31.5	35	32.4	11	28.9
Grade II	13	8.9	12	11.1	1	2.6
Grade IIIa	2	1.4	2	1.9	0	0
Grade IIIb	1	0.7	1	0.9	0	0
Grade IV–V	0	0	0	0	0	0
Total	146	100	108	100	38	100

Values are presented as absolute numbers and percentages. Percentages in the total cohort column are calculated based on the full study population (*n* = 146), while percentages in the on-clamp and off-clamp columns are calculated within each surgical subgroup. Grade 0 indicates absence of postoperative complications. ≥Grade II and ≥Grade III represent clinically significant complications according to the Clavien–Dindo classification system.

**Table 5:** Clavien–Dindo postoperative complications stratified by surgical approach (on-clamp vs. off-clamp RAPN) with comparative analysis of clinically significant complications (≥Grade II and ≥Grade III), including effect estimates and statistical testing.

Outcome	On-Clamp	Off-Clamp	OR (95% CI)	*p*-Value
≥Grade II	15/108	1/38	5.97 (0.91–64.67)	0.0705
≥Grade III	3/108	0/38	Not estimable	0.5679

**Table 6:** Trifecta outcomes in evaluable patients with malignant renal tumors (*n* = 130).

Parameter	Category	Total (*n* = 130)	%	On-Clamp RAPN (*n* = 99)	%	Off-Clamp RAPN (*n* = 31)	%
Trifecta outcome ^‡^	Achieved	120	92.3	90	90.9	30	96.8
	Not achieved	10	7.7	9	9.1	1	3.2
Effect size (OR)	On-clamp vs. Off-clamp	–	–	0.326	–	–	–
95% CI (OR)	Baptista–Pike method	–	–	0.029–2.195	–	–	–
*p*-value	Fisher’s exact test	–	–	0.4498	–	–	–

^‡^ Trifecta was defined as the simultaneous achievement of negative surgical margins, warm ischemia time ≤ 25 min (not applicable in off-clamp procedures), and absence of Clavien–Dindo grade ≥ III complications within 3 months postoperatively. Group denominators were *n* = 99 for the on-clamp group and *n* = 31 for the off-clamp group. Percentages are calculated within each surgical subgroup.

## Data Availability

The datasets generated and analyzed during the current study are available from the corresponding author upon reasonable request for academic or research purposes.

## Supplementary Materials

The following supporting information can be downloaded at: https://www.mdpi.com/article/10.3390/diagnostics16101543/s1, Table S1. STROBE Checklist for Observational Studies.

## Abbreviations

The following abbreviations are used in this manuscript:

Cr	Creatinine
EBL	Estimated blood loss
eGFR	Estimated glomerular filtration rate
Hb	Hemoglobin
PN	Partial nephrectomy
RAPN	Robot-assisted partial nephrectomy
RCC	Renal cell carcinoma
RN	Radical nephrectomy
SD	Standard deviation
SSIs	Surgical site infections
