# Supplementary material for: On-Clamp Versus Off-Clamp Robot-Assisted Partial Nephrectomy for Localized Renal Tumors: A Retrospective Single-Center Cohort Study
Source: Diagnostics (Basel). 2026 May 19;16(10):1543. doi: 10.3390/diagnostics16101543 (PMC13206001; doi:10.3390/diagnostics16101543)
Supplement: Supplementary file 1 [file diagnostics-16-01543-s001.zip › diagnostics-4267262-supplementary.pdf]

Supplementary Table S1. STROBE Checklist for Observational Studies

Section	Item No.	Recommendation	Reported on Page No.
**Title and Abstract**	1	Indicate the study's design with a commonly used term in the title or abstract	Title; Abstract
	2	Provide an informative and balanced summary of what was done and found	Abstract
**Introduction**	3	Explain the scientific background and rationale	Introduction
	4	State specific objectives and hypotheses	Introduction (end)
**Methods**	5	Present key elements of study design early in the paper	Section 2.1
	6	Describe the setting, locations, and relevant dates	Section 2.1
	7	Describe participants, eligibility criteria, and selection methods	Section 2.1
	8	Define all variables (outcomes, exposures, confounders)	Sections 2.2–2.4
	9	Describe data sources and measurement methods	Sections 2.2–2.4
	10	Describe efforts to address potential sources of bias	Sections 2.1; Discussion
	11	Explain how the study size was determined	Section 2.1
	12	Explain handling of quantitative variables	Section 2.7
	13	Describe statistical methods, including confounding control	Section 2.7
	13a	Describe methods used to control confounding	Section 2.7
	13b	Describe subgroup analyses	Section 2.7
	13c	Explain handling of missing data	Section 2.7
	13d	Describe sensitivity analyses (if applicable)	Not applicable
**Results**	14	Report numbers of individuals at each stage of study	Figure 1
	15	Describe characteristics of study participants	Section 3.1
	16	Report numbers of outcome events or summary measures	Sections 3.3–3.4
	17	Report main results (estimates and precision)	Section 3
	18	Report other analyses (subgroups, interactions)	Section 3
**Discussion**	19	Summarize key results	Discussion
	20	Discuss limitations, including bias	Discussion
	21	Provide interpretation considering objectives and limitations	Discussion
	22	Discuss generalizability	Discussion
**Other Information**	23	Funding sources and role of funders	End of manuscript

Note: This checklist follows the STROBE (Strengthening the Reporting of Observational Studies in Epidemiology) Statement.
